# Bioinspired self-healing materials: lessons from nature

**DOI:** 10.3762/bjnano.9.85

**Published:** 2018-03-19

**Authors:** Joseph C Cremaldi, Bharat Bhushan

**Affiliations:** 1 Nanoprobe Laboratory for Bio & Nanotechnology and Biomimetics (NLBB), The Ohio State University, 201 W. 19th Avenue, Columbus, Ohio 43210-1142, USA

**Keywords:** animals, biomimetics, bioinspired, capsules, functional coatings, healing mechanisms, plants, protective surfaces, self-healing, vascular systems

## Abstract

Healing is an intrinsic ability in the incredibly biodiverse populations of the plant and animal kingdoms created through evolution. Plants and animals approach healing in similar ways but with unique pathways, such as damage containment in plants or clotting in animals. After analyzing the examples of healing and defense mechanisms found in living nature, eight prevalent mechanisms were identified: reversible muscle control, clotting, cellular response, layering, protective surfaces, vascular networks or capsules, exposure, and replenishable functional coatings. Then the relationship between these mechanisms, nature’s best (evolutionary) methods of mitigating and healing damage, and existing technology in self-healing materials are described. The goals of this top-level overview are to provide a framework for relating the behavior seen in living nature to bioinspired materials, act as a resource to addressing the limitations/problems with existing materials, and open up new avenues of insight and research into self-healing materials.

## Review

### Introduction

The ability to heal is intrinsic to all multicellular organisms. Every organism has evolved to occupy a specific role in the ecosystem, with underlying themes in reproduction, animal complexity, the food chain, and the environment [1–3]. Evolution has created a very large amount of diversity in the animal and plant kingdoms. Approximately 1 M of the 7.7 M animals thought to exist have been discovered [1,4–5], and on the order of 200,000 out of the 300,000 plant species thought to exist have been discovered [4–5]. This biological diversity has also resulted in incredibly diverse types of healing and injury prevention found throughout nature. Therefore, having a clear and bounding definition of healing is paramount.

**Figure 1:**
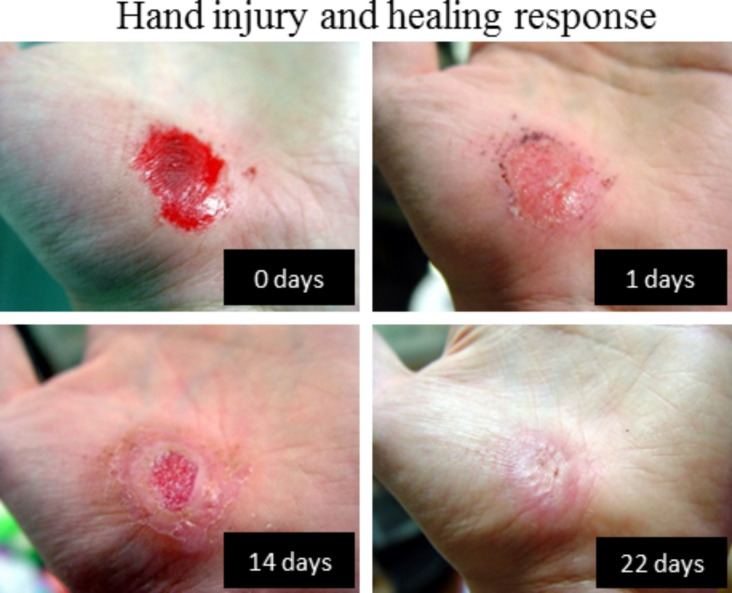
Photographs of a hand injury healing over time. The pictures show the immediate need for a healing response to stop blood loss all the way through remodeling and scarring after 22 days. Adapted from [7].

The Merriam-Webster dictionary [6] defines healing as the ability “to make free from injury or disease - to make sound or whole.” The hand injury seen in Figure 1 shows the healing process in action, where the body stops the bleeding and then regrows tissue over the wound over a longer timescale [7]. Other definitions account for the extent of healing. Reference [8] defines healing as “a phenomenon consisting of sequentially controlled steps which result in the replacement of dead tissue with regenerated cells and/or scar tissue” and [9] states that regenerative medicine “…seeks to repair or regenerate damaged tissue and organs…without leaving scar tissue behind, thereby restoring both structure and function of tissues/organs.” The overall goal of life is to survive, and healing is one of the tools an organism uses to achieve this goal. Healing can occur through regeneration, repair, or replacement of damaged tissue, noting that to regenerate is to repair 100% of the damage, that is, the ideal form of repair is regeneration. With these ideas in mind, one can look at the ways different organisms heal.

Evolution has provided every organism with healing mechanisms to fight for survival against injury and infection. In general, vertebrates (animals with a backbone) are more biologically complex animals than invertebrates (animals without a backbone), which is matched by complexity in healing. Invertebrates tend to have shorter lifetimes and high reproduction rates. In comparison, vertebrates tend to have long lifecycles and the lowest reproduction rates in the animal kingdom; each individual vertebrate’s survival is important for the survival of the species.

The plant kingdom has also produced many complex types of healing. As the base of the food chain, plant life is varied and robust to ensure widespread growth and high reproductive rates [10–11]. In some ways, plants and animals approach healing in the same ways, including the existence of immune systems and damage prevention. However, the differences in need and biology between plants and animals creates unique pathways as well, such as damage containment in plants and clotting in animals. Damage containment describes the process of sealing away or discarding infected/damaged tissue. For instance, losing a tree limb does not affect the fate of the tree in the same way that losing a leg would affect the survival of a gazelle.

The aim of this review is to understand healing and the defense mechanisms seen in living nature and apply them to bioinspired approaches in engineering. While individual cells and single-celled organisms have the ability to heal [12], they mainly rely on high reproductive (cell division) rates and short life spans (fast evolution) for proliferation and survival. This review focuses on the multicellular plant and animal organisms, whose healing goal is homeostasis, or the body’s attempt to maintain itself. Homeostasis is defined as a dynamic steady state in the internal environment.

We first look at the varied healing and defense mechanisms seen across the plant and animal kingdoms. Once these various mechanisms have been described, the following section categorizes the mechanisms and provides a prevalent example of each. Next, we transition from animal and plant mechanisms to bioinspired self-healing materials, providing examples and description of prominent approaches and connecting each with its roots in living nature. Last, we provide conclusions and outlooks.

### Self-healing and defense mechanisms found in living nature

This section focuses on defense and healing in multicellular organisms. We first look at animals (fauna) and then plants (flora). Table 1 summarizes our organizational approach to describe healing in such a large and diverse data set. The two major categories of organisms, fauna and flora, will be further divided into subsections, as seen in the first column of Table 1. Fauna will be broken down by vertebrates, animals with a spinal cord, and invertebrates, those animals without a spinal cord or skeleton. Additionally, healing will be broken into soft tissue wounds and hard tissue (e.g., bone or exoskeleton) wounds for both vertebrates and invertebrates. Flora will be broken down into herbaceous and woody plants. Herbaceous plants die down to the ground each year and regrow (perennials, annuals, and biennials), whereas woody plants refer to trees and plants that maintain a persistent woody stem above ground year round. The second column in Table 1, “Physical change,” describes the range of physical changes that organisms may go through either voluntarily or after being wounded. With the definition of healing in mind, returning to an original form/function by making sound or whole, the column that is labelled as “Healing response/defense description” describes an organism’s response to the physical changes in healing itself. The “Mechanism(s) in living nature” column breaks the healing and defensive responses down into simple mechanisms and most basic functions found in living nature.

**Table 1:** Self-healing and defense mechanisms found in living nature.

	Physical change	Healing response/defense description	Mechanism(s) in living nature	Ref.

Fauna				

Vertebrates and invertebrates	Muscle contraction and relaxation	Proteins actin and myosin react with one another to reversibly slide against each other	Reversible contraction and relaxation	[13]
	Adaptive camouflage	Physical shape changes in skin can change light reflection or control pigmentation depth in the skin	Muscle control or swelling of skin to control light reflectivity or pigmentation	[14] [15]
	Innate immune response	Anatomical barriers, fluids (tears or mucus), cytokines and phagocytes	Physical barriers and cellular response to harmful microbes	[16]
	Central nervous system (CNS) injury	Cellular response: inflammation, blood–brain barrier reestablishment and axonal breakdown, and limited axon regeneration after glial scarring	Clearing of debris and rapid system stabilization to limit damage	[17] [18]
	Peripheral nervous system (PNS) injury	Cellular response: inflammation, Schwann cells and macrophages clear debris, and axonal growth reconnects the proximal and distal nerve segments	Clearing of debris with limited scarring, allowing for cell regrowth	[17] [18]
Vertebrate hard tissue	Bone break	Cellular response: inflammation, cartilage callus formation, lamellar bone generation and remodeling	Cellular response to remove debris, stabilize injury, and remodel (heal) the wound	[19]
Vertebrate soft tissue	Wound	Cellular response: clotting, inflammation, proliferation, and remodeling	Cellular signaling from short-term clotting to long-term cell growth and remodeling	[20]
	Stem cell response	Stem cells replicate and can differentiate into all cell types in order to produce new tissue	Adapted healing through regrowth of tissue	[21] [22]
	Molting (shedding) and replenishment	Discarding and replacing old, dead tissue to accommodate new growth (reptiles) or seasonally (birds, cats)	Shedding of the outermost layer (skin, feathers, hair)	[23] [24]
	Adaptive immune response	Immunological memory: antigen recognition, tailored cellular response, and B,T-cell memory	Infection/disease-specific cellular response triggered by the innate immunity	[25]
Invertebrate hard tissue	Wound	Endoculticle secretion from the cellular epidermis	Continual growth of exoskeleton from within, hard outer layer protecting soft inner layer	[26]
	Exoskeleton growth and ecdysis	Shell replacement as organism outgrows its current exoskeleton	Periodic replacement of outer protective exoskeleton with new growth	[17]
Invertebrate soft tissue	Wound	Clotting through plasma protein reactions, blood cell aggregation, or cell population explosion followed by cellular healing	Clotting through dense hemocyte network, hemocyte/plasma coagulation, cell reproduction	[27] [16]

Flora				

Herbaceous and woody plants	Cell walls	Barrier between plant cells that can serve as barriers to pathogens or enzymatic degradation	Physical barriers and segmentation	[28]
	Wound closure and growth	Seal-off and grow: production, differentiation, and maturation of the callus parenchyma	Cells swell and divide through hypertrophy/hyperplasia and then harden at the surface	[29]
	Secretions after injury	When breached, special secretion cells release latex, gum, or volatile oils to aid healing/defense	Special cells are punctured and release a localized response	[30] [31]
	Self-cleaning	Epicuticular wax offers water-loss protection and self-cleaning properties	Replenishable functional coatings create beneficial surface behaviors	[28] [32]
	Active abscission or shedding dead tissue	Hydrolytic enzymes degrade cell walls in a separation layer to shed leaves, fruit, etc.	Enzymatic degradation of cell wall adhesion before abscission and scarring after	[33]
	Innate immune response	Pattern-recognition of microbe attack to initiate an appropriate response	Cellular response for specific cell response	[34]
Woody plants	Growth of bark on the tree (epidermis) and roots (rhizodermis)	Continual production of thick outer layers of the epidermis (tree bark) and rhizodermis (root bark) protecting inner layers of plant and root systems	Continual replenishment of the hard, protective layers	[35] [36] [28]
	Compartmentalization of decay in trees	Damaged tissue sealed chemically from undamaged tissue to prevent the spread of decay	Boundary formation to isolate injured tissue	[37] [38]

#### Fauna

All animals share many basic characteristics, including chemical uniqueness, complexity and hierarchical organization, reproduction, genetic program (DNA), metabolism, developmental life cycle, environmental interaction, and movement [25]. However, one major division in the animal kingdom that changes the way in which animals heal or grow is vertebrates versus invertebrates. Vertebrates possess an internal skeleton and/or spinal cord to provide structure and transfer the forces generated by muscle [2]. Invertebrates are animals with an exoskeleton or no skeleton at all. In terms of population, invertebrate species make up about 95% of all species, vastly outnumbering their vertebrate counterparts [1,5]. Well-known invertebrate species include insects, crustaceans, snails, clams, octopuses, spiders, jellyfish, starfish, worms, and coral. Within the invertebrates, the subset of arthropods is of particular interest and accounts for approximately 85% of species variation [1–2,5]. Arthropods are characterized by segmented bodies, exoskeletons, and appendages occurring in pairs. This includes all insects, arachnids (spiders), myriapods (e.g., millipedes and centipedes), and crustaceans (e.g., crabs and shrimp) [25].

Using the common characteristics of all animals, and the physical differences between vertebrates and invertebrates, one can see where similarities and differences exist in their healing and growth. The similar, specialized cell and tissue systems include muscles (movement), an immune system, and a nervous system. Although the complexity of such systems differs between vertebrates and invertebrates, the mechanisms through which they heal are similar throughout, and the healing responses that are common to both vertebrate and invertebrates will be discussed first. Afterwards, we look at the responses specific to vertebrates and then invertebrates. These cell and tissue systems include circulatory and skeletal components that depend on the type of circulatory and skeletal systems. Vertebrates have an internal skeleton (endoskeleton) and use a closed circulatory system with blood running through channels (veins and arteries) throughout the body. Invertebrates have an exoskeleton or no skeleton and typically have an open circulatory system with hemolymph (the equivalent of blood) running throughout the body without restriction. In light of these characteristics, the vertebrate and invertebrate subsections will be further broken into respective soft tissue and hard tissue healing responses.

**Figure 2:**
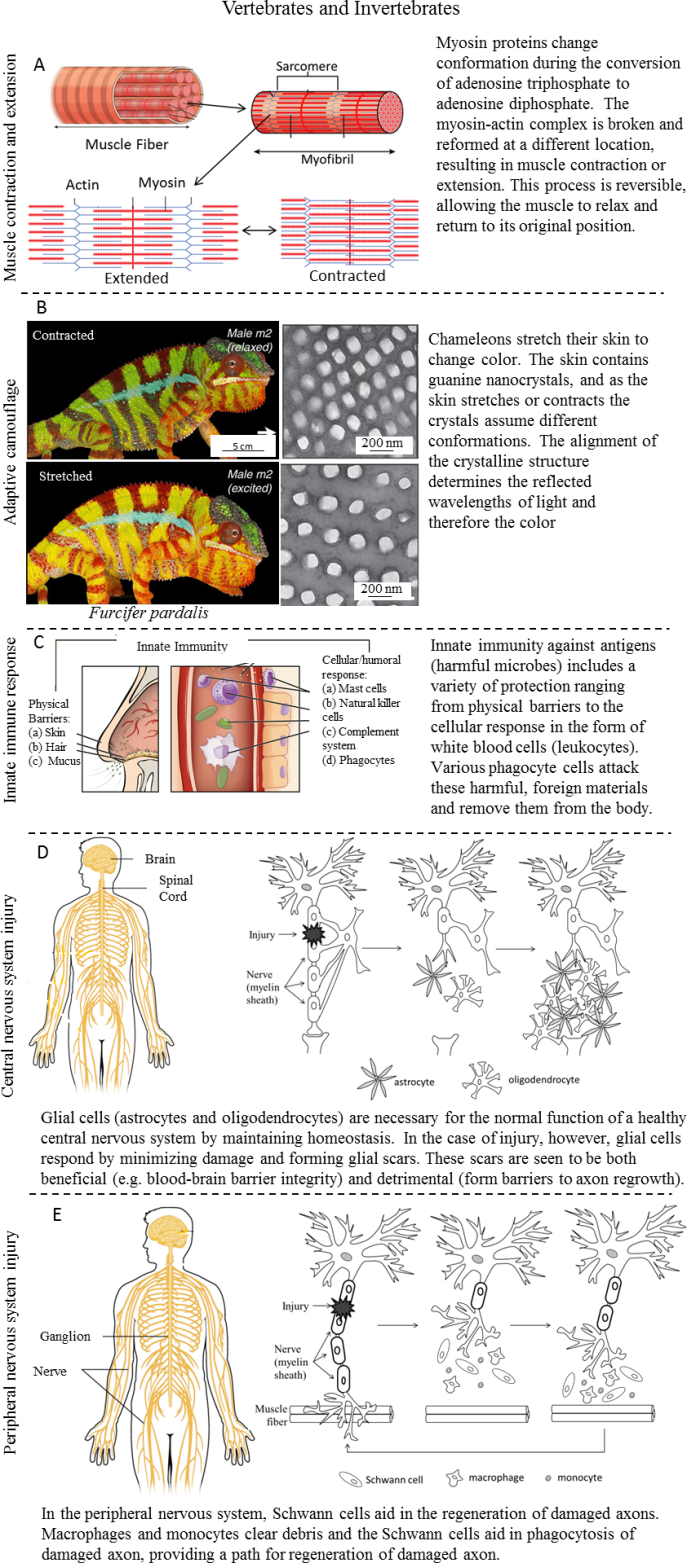
Healing and defense mechanisms shared by vertebrates and invertebrates including (A) muscle extension and contraction, (B) adaptive camouflage, (C) innate immunity, and (D, E) healing of central and peripheral nervous system injuries. (A) The hierarchical structure of muscle is shown, building up from the basic protein components of actin and myosin. Adapted from [39]. (B) Color change in chameleons occurs through reversible muscle control of guanine nanocrystals in their skin. Adapted from [15]. (C) Innate immunity consists of physical barriers to harmful microbes as well as an internal cellular and humoral response should they gain access to the body. Adapted from [40]. (D) In healing of a central nervous system injury, glial cells in the extracellular matrix quickly form scar tissue to maintain homeostasis, but may prevent axon repair in the process. (E) In healing a peripheral nervous system injury, Schwann cells clear the area around the axons and allow for repair without obstruction. The left images in (D) and (E) were adapted from [41].

**Vertebrates and invertebrates:** This section analyzes healing and defense mechanisms common to both vertebrates and invertebrates (Figure 2). When looking at characteristics that transcend the animal kingdom, perhaps the most obvious common physical change is reversible movement. All animals, even sponges, move in some way [2]. Adaptive camouflage, while not characteristic of every animal, can be found in both vertebrates and invertebrates. It is a type of muscle movement that has been singled out due to the controlled ability to change a physical characteristic of the animal. Other complex cell and tissue systems that will be described include the immune response to harmful microbes/injury and the possession of a nervous system. As previously mentioned, vertebrates tend to be more complex and therefore have more complex systems. This complexity affects our breakdown of both the immune system and the nervous system. All animals have the innate immunity that is covered in this section. Adaptive immunity, present only in vertebrates, is covered later. Invertebrate nervous systems are very simple. While all animals have a nervous system, it is more difficult to differentiate between the (central and peripheral) nervous system components in invertebrates. Therefore, we focus on the more complex healing in a vertebrate (human) nervous system to help differentiate between the CNS and PNS.

*Muscle contraction and extension* – Every animal uses muscle to convert chemical energy into mechanical energy. Types of muscle include skeletal, smooth (striated), and cardiac muscle, which only exists in vertebrates. These divisions are based on characteristics of a muscle’s usage/location, involuntary/voluntary control, and cell size/shape [14]. Figure 2A shows the hierarchical levels of muscle structure in a human, building from the basic components of actin and myosin proteins to sarcomeres and muscle fibers that form a muscle [39]. Skeletal muscle is striated and used for voluntary movements. Smooth, or nonstriated, muscle is used for involuntary muscle movements such as breathing and digestion. Last, cardiac muscles are involuntary, striated muscles used in the movement of the heart and are organized into a complex linkage between sarcomere fibers. Invertebrates have several types of muscles related to body function such as digestion, locomotion, or flight. Unlike vertebrates, however, all insect muscle is striated and under voluntary control [17].

Despite the variation in characteristics and usage, all muscle is dependent on the interaction between the proteins actin and myosin at the most basic level. A great deal of research has been put into the current understanding of muscle movement, as described by [42]. Through regulation and interaction, these two proteins slide past one another, causing a reversible extension/contraction and then relaxation of muscle on a macroscale, as detailed by [43]. This mechanical interaction between actin and myosin is fueled by the energy loss in adenosine triphosphate (ATP) conversion to adenosine diphosphate (ADP) [13,44]. This process is regulated by the free Ca^2+^ concentration in the extracellular matrix, as described by Cooper [45]. Three regulatory mechanisms have been identified: Ca^2+^ binding to troponin-tropomyosin, phosphorylation of myosin by a (Ca^2+^) dependent kinase, and direct binding of the free Ca^2+^ to myosin. When regulated/deregulated the globular portion of the myosin protein attaches/detaches from the actin filament, causing the sliding motion.

To sum up, when looking at the healing nature of muscle movement, it is important to note the reversible nature of the movement. In other words, every animal has the ability to return to form and function after every movement (contraction and relaxation) through the transformation of (chemical) energy to a mechanical force.

*Adaptive camouflage* – Adaptive camouflage is a specific use of muscle control to change color. This change allows an organism to blend in with their immediate surrounding. The four methods of coloration shading used for adaptive camouflage are color resemblance, obliterative shading, disruptive coloration, and shadow elimination [14]. Adaptive camouflage may be used for avoidance of predators, to enhance visibility during courtship, to exert dominance over competitors, or as a preemptive warning to predators.

To accomplish these types of color changes, an organism needs to change the way light reflects off of its skin. Skin contains chromatophores, or pigment-containing cells, which can change in two ways. First, the chromatophores can change chemically with season, diet, etc. to a new color. In a more direct second method, some animals control their skin coloration through muscle control. Figure 2B shows an example of a chameleon that, by stretching its skin, can change the spacing and conformation of guanine nanocrystal lattices embedded in its skin [15]. In a similar fashion, some animals can use muscle control to disperse or aggregate chromatophores in the dermal layer, changing their appearance. In both of these muscle-controlled cases, animals have control over a reversible process to change a physical property.

To sum up, adaptive camouflage allows an animal to change color through muscle control. The important aspect of the camouflage is that the change is controlled and the animal can return to its original function/form (color in this instance).

*Innate immune response* – Immunity refers to the “global ability of the host to resist the predation of microbes that would otherwise destroy it” [46]. This resistance to harmful microbes exists as a natural (innate) ability existing in all animals, vertebrate and invertebrate alike. Learned (adaptive) immunity exists only in vertebrates and will be discussed in a later section.

Innate immunity consists of physical barriers and an internal response to antigens that make it past those barriers. Figure 2C shows an example of an innate immune system in a human, with external defenses such as hair, skin, and mucus and internal defenses such as mast cells, natural killer cells, and phagocytes [40]. The internal response in all animals has both humoral and cellular components [47]. Humoral components refer to those existing in the extracellular matrix, such as proteins in the complement system, antibacterial agglutinins, and antitoxins. The extracellular matrix is the collection of molecules and secreted materials outside of cells that is tasked with providing support structurally and chemically to the cells in their immediate area.

The cellular component of the innate immune system refers to the leukocytes, white blood cells, tasked with responding to harmful microbes [14]. Each of these cell types has a specific duty. Mast cells control the inflammatory response in a wound by secreting/releasing specific compounds. Natural killer cells look for foreign antigens and then proceed to attack them. A more specific cellular response is triggered when the complement cascade, using proteins formed in the liver, marks foreign cells for removal or attack. Once marked, phagocytes kill and remove these foreign cells.

Invertebrates have a very similar innate immune response. In comparison to the skin, hair, or feathers of vertebrates, invertebrate physical barriers mainly consist of an exoskeleton such as mollusk cockle, sea urchin test, and arthropod cuticle. In the absence of an exoskeleton (e.g., an octopus), barriers include mucus, melanin, and/or agglutinin. The cellular response of invertebrates includes haemocytes/coelomocytes (invertebrate equivalents of blood cells and leukocytes) circulating throughout the body, humoral factors, and complement factors to aid in the destruction and removal of harmful microbes [16,48]. Comparative studies of invertebrate immune systems in relation to vertebrate immune systems show the simplicity of invertebrate systems, and hence may offer insight into the complex systems of vertebrates.

To sum up, all animals have an immune defense system to protect the organism from harmful microbes. This defense consists of two layers. The first consists of protective physical barriers to prevent the damaging microbes from entering the body. The second line of defense is the internal, humoral and cellular ability to both recognize and remove these harmful microbes.

*Central nervous system (CNS) injury* – All multicellular animals (except sponges) have a nervous system controlling voluntary and involuntary actions [17]. The nervous system can take a variety of forms depending on the cerebral ability and necessary complexity of an animal. In vertebrates, the nervous system has the job of processing/filtering sensory signals, introducing memories, and sending out an appropriate response. This highly complex sequence of events relies on nerve cells, or neurons, running throughout the body (Figure 2D,E). This branching network of nerves, where dendrite nerve cells gather into axons, connects through the spinal cord to the brain, the central processing part of a body. The combination of the spinal cord and brain is what makes up the central nervous system (CNS). In (simpler) invertebrates, the range of organisms has led to the evolution of several alternative simpler central/peripheral nervous system designs including nerve nets, segmented systems, and nerve ladders as described by Moffett [49]. In fact, the most simple nervous system designs (flat worms, sea cucumbers, and ribbon worms) can regenerate as long as enough neural tissue remains to stimulate regeneration [49]. It is believed that these more basic systems are the evolutionary precursors to the more complex systems seen in vertebrates, lending towards their usage as model systems for study [50].

This review uses a human nervous system as the example in Figure 2D,E [41]. Following nervous system injury, enough of this system must remain intact to maintain homeostasis and initiate healing. A healthy CNS relies on glial cells in the extracellular matrix to maintain homeostasis. Due to the importance of the CNS, injury creates competing needs of (1) survival through maintaining homeostasis and (2) regeneration of the nerves. The glial cells (e.g., astrocytes and oligodendrocytes) exist to protect neurons and maintain homeostasis by removing material and minimizing damage to the body. However, this “protection” of the blood–brain barrier through glial scarring also creates barriers in the exact area where axons need to regrow, in effect limiting the healing response in the CNS [18,51].

To sum up, in vertebrates the CNS component of the nervous system is highly protected through both the spinal column and glial cells which are meant to maintain the homeostatic environment surrounding the nerves. In case of injury, however, the need to maintain homeostasis competes with the need to heal, resulting in rapid scarring from the glial cells. While the scarring helps to ensure survival, it can lead to limited nerve healing due to the blockage in nerve pathways by those scars.

*Peripheral nervous system (PNS) injury* – The peripheral nervous system (PNS) consists of the branching network of nerves that attaches to the receptors sending signals to the brain or sending signals to muscle from the brain (Figure 2E). Injuries to the PNS tend to have more restorative function after healing than in the CNS. The reason is the body’s less drastic response in dealing with the injury. As survival (typically) is not as much in question after a PNS injury, the healing response lends for a more restorative path. Macrophages and monocytes, types of white blood cells from the inflammatory immune response, clear material from the wound site. Schwann cells are a type of glial cell particular to the PNS and its healthy homeostatic function. With the debris cleared, Schwann cells are free to remove damaged axons and help reconnect nerves in axonal growth [18,51].

To sum up, the PNS, while similar to the CNS, elicits a much different cellular response. The cells meant to maintain homeostasis around the PNS, Schwann cells, and clear away material to help neuron regrowth (rather than scarring as is seen in the CNS) and restore function.

**Vertebrate hard tissue:** Hard tissue in vertebrates refers to bone. Bones are the porous and mineralized structure that creates the skeletal system. They house the marrow, nerves, and blood vessels that make up vital systems to homeostasis and ensure regular function of vertebrate bodies [52]. When a bone break occurs, both soft tissue damage (in encased blood vessels) and nerve damage occur as well. However, this section focuses directly on the remodeling of the bone itself, with soft tissue and nervous system healing covered in other sections.

**Figure 3:**
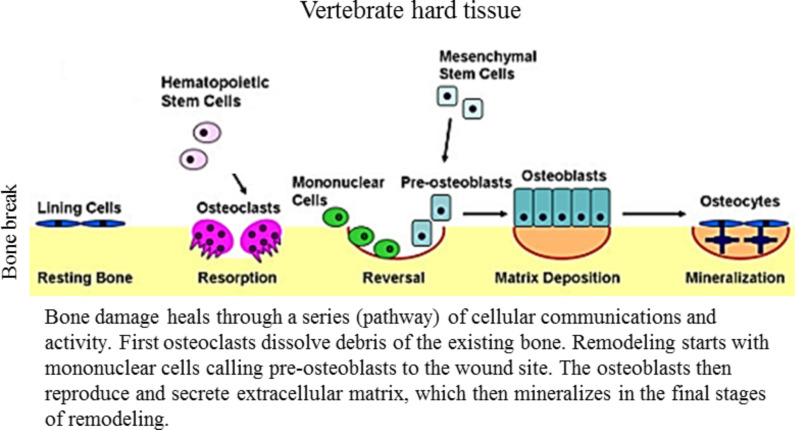
Healing in vertebrate hard tissue showing the stages of bone tissue repair. Healing stages include mechanisms to stabilize the sides of the bone injury until the remodeling phase completes, restoring form and function to the bone tissue. Adapted from [53].

A bone injury heals through a complex set of cellular signals and humoral responses as can be seen in Figure 3 [53]. These responses are meant to provide both short-term and long-term “fixes” to the wound, eventually ending with remodeling (restoration) of the bone to its original form as detailed by Crockett et al. [19]. Immediately following a bone injury, cells in the immediate area begin to secrete chemicals and initiate a chain of healing events meant to minimize pain, remove material, and rebuild the bone. The immediate response is directed towards the immune and soft tissue, including clotting, which stems the flow of blood, and inflammation, where increased blood flow brings phagocytes. The short-term bone response is chondroblasts, which replicate themselves and secrete extracellular matrix that includes collagen. Collagen is a strong structural protein that forms the cartilage callus, which holds the bone stationary while the remodeling phase occurs. Remodeling entails the phase wherein osteoclasts, large and multinuclear cells, resorb bone at the wound site and osteoblasts begin to secrete new bone matrix. This new bone matrix eventually forms mechanically strong lamellar bone, which then mineralizes to end the healing process. In an ideal remodel, the setting of the bone during healing causes the two fragments or sides of the wound to line up perfectly. In reality, however, material filler, scar tissue, fills in any gaps when the wound heals improperly.

To sum up, the healing of vertebrate hard tissue, bone, encompasses a complex set of cellular responses. These responses are meant to provide both short-term and long-term healing. The short-term healing stops the damage and maintains a weaker structure to hold the bone in place until the long-term and full strength bone remodeling finishes.

**Figure 4:**
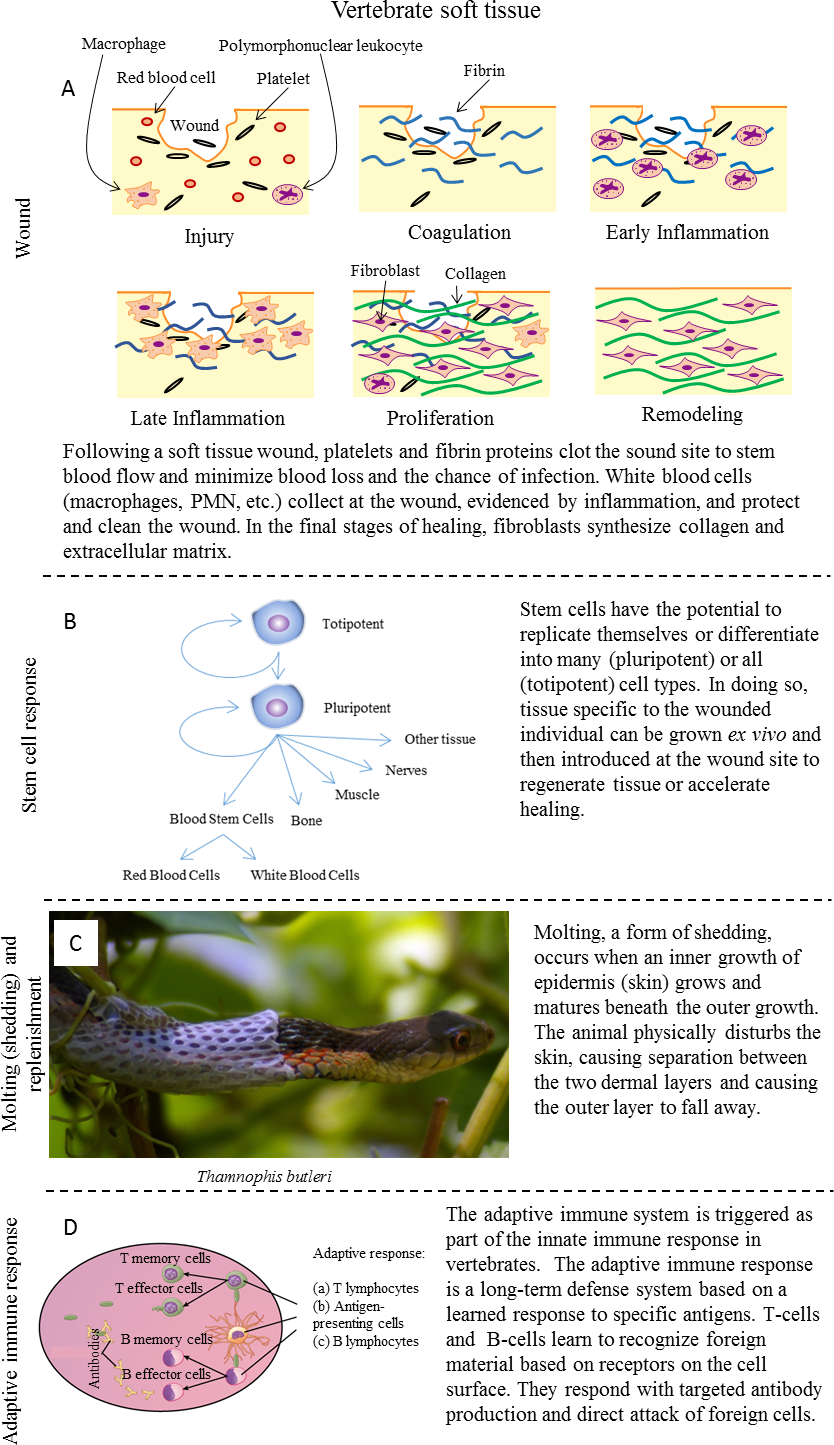
Types of healing in vertebrate soft tissue is shown, including (A) wounds, (B) stem cell differentiation, (C) shedding, and (D) the adaptive immune system response. (A) Soft tissue wounds elicit a short-term clotting response followed by a long-term cellular signaling response ending with remodeling of the damaged tissue. Adapted with permission from [54], copyright 2003 Cambridge University Press. (B) Stem cells, the original cells that all other cells develop from, offer unique healing opportunities through replication and differentiation. (C) Some vertebrates periodically shed their epidermal tissue for growth or seasonal changes, revealing new tissue beneath. Reproduced from [55], copyright 2009 Goellnitz, under CC-BY-NC 2.0 license (https://creativecommons.org/licenses/by-nc/2.0/). (D) The adaptive immune system, unique to vertebrates, utilizes a stored “memory” response to fighting and removing harmful microbes. Adapted from [40].

**Vertebrate soft tissue:** Similar to a hard tissue wound, soft tissue healing encompasses an immediate response and long-term remodeling phase. Singer and Clark [20] detail these phases of healing in a soft tissue wound injury, which are shown in Figure 4A [54]. Immediately after an injury to vertebrate soft tissue, platelets gather at the site of the wound to stem the loss of blood while also preventing foreign and potentially harmful material from entering the body. Inflammation initiates the innate immune response, often seen as a swelling redness as an increase in cells occurs at the site of the wound. In proliferation, fibroblasts (counterparts to the osteoblasts in hard tissue) secrete extracellular matrix, fibrinogen, and collagen to remodel tissue at the site of the wound. Over time, dermal tissue layers reform, eventually creating a new skin layer. Misalignment in the healing and remodeling process shows as a scar once the repair ends.

To sum up, the soft tissue healing response in vertebrates has a short-term component and long-term component similar to the hard tissue response. In soft tissue, the short-term response involves clotting of the wound to stem blood flow. Over a longer period, the cellular response leads to remodeling of the epithelial (skin) tissue at the wound site.

*Stem cell response* – In the discussion between repairing damaged tissue versus the ability to regenerate damaged tissue (100% repair), stem cells are the focal point. Regeneration can be found in invertebrates such as hydras and flatworms [56] or in vertebrates such as salamanders [57]. On a smaller scale, hair, skin, and blood cells all regrow to be exact replicas of the lost tissue in a cycle known as physiological regeneration [58]. However, humans have long studied regeneration with the aim of expanding the concept to larger parts of the body [57], aiming to reproduce the effect of regenerative abilities in salamanders [59]. This idea has been more of a “myth and a dream” [60], with only the recent use of stem cells promising results on a smaller scale [61].

Stem cells refer to the original cells in an organism that replicate and mature (differentiate) to create every part of the organism. These cell types include skin, blood, muscle, and nerve cells as seen in Figure 4B [62]. Additionally, stem cells have the potential to expedite the healing process and are completely biocompatible when unique to the individual [21–22]. Stem cells in their most basic, embryonic form are referred to as undifferentiated or totipotent, having the potential to become all other cell types. By differentiating, embryonic stem cells can turn into partially differentiated, tissue-specific cells. A blastocyst, a cell mass and source of embryonic stem cells, contains all of the information and material necessary to create a complete being. As the blastocyst differentiates, it loses parts of its potency, becoming pluripotent. The differentiation pathway decisions from the cell depend on several environmental characteristics, including mechanical stresses, the chemical environment of the extracellular matrix, and chemical activity and (cell–cell) interactions with neighboring cells. In this way, stem cells change to whatever is needed at the site of an injury to help restore form and function [21].

To sum up, stem cells are the embryonic material that other cells form from. In their most basic form, they hold the ability to differentiate into every other cell in a body. If grown outside of the body, this offers a unique and expedited chance at healing and regeneration when introduced at the site of a wound. They differentiate through stress, conditions in the extracellular matrix, or cellular signaling.

*Molting (shedding) and replenishment* – In vertebrates, a large number of biological functions rely on the shedding of dead tissue and its replacement with healthy, new tissue. Molting occurs as a form of shedding and can include layers of skin, hair, and/or feathers. Figure 4C shows a well-known example of molting which occurs when a snake sheds it outer skin [55]. Unlike molting that occurs simply for hygiene and/or seasonally, snakes and other organisms may simply outgrow their skin and need to shed (slough) to grow in size [24,63]. In this process, a new skin layer, the inner growth (IG), grows beneath the older skin, the outer growth (OG). Once the new growth has matured enough to function properly in the survival of the animal, the outer layer is shed off to reveal the new skin layer. In the case of the snake, physical movement and rubbing are used to create the separation between the two layers of skin. Maderson [23] breaks the process into six distinct phases: (1) the bright new skin found immediately after sloughing, (2) the stratum germinativum forms a new presumptive inner epidermal generation between itself and the outer epidermis, (3) inner epidermal cells begin to keratinize, (4) new tissue layer formation between the inner and outer epidermal regions (stratum intermedium), (5) lacunar tissue breakdown in the stratum intermedium, and (6) sloughing of the keratin layer.

To sum up, some animals require shedding of their skin to grow. This replacement occurs through a type of layering in which a new skin is grown beneath the old, eventually forcing it off and replacing it. This growth from within process repeats throughout the life of an animal.

*Adaptive immune response* – The adaptive immune response is triggered by and is subordinate to the innate immune response in vertebrates [46]. The main cellular components of adaptive immunity are lymphocytes, a subset of leukocytes (white blood cells). T-cells and B-cells, leukocytes shown in Figure 4D, each offer a unique approach to sterilizing harmful microbes [40]. These cells exist as short-term response effector cells or as (response) memory cells. They are regulated by the complement cascade of the innate immune system [64]. T-cells come from the thymus and use a cell-mediated immune approach by lysing (destroying the cell membrane of) infected cells or causing apoptosis (induced/programmed cell death) in infected cells [25]. B-cells originate in bone and create the humoral path of adaptive immunity by creating antibodies, large proteins able to recognize receptors in harmful microbes. After locking on to the harmful microbe, antibodies either mark them to be destroyed or block their active regions, effectively neutralizing them [25]. Vaccination (controlled exposure) to pathogens and surviving an illness create the immunological memory necessary for these tailored responses. More recently, an argument has been made to include natural killer (NK) cells in the adaptive response as well [65]. NK-cells serve a similar purpose as T-cells, but operate on a shorter timescale by recognizing cells under “stress” without the need for receptor recognition.

To sum up, the key concepts from adaptive immunity are that vertebrates have a cell-memory system to recognize harmful microbes and produce a targeted, specific response to resist and remove them. This memory response is based on the ability of B-cells and T-cells to recognize and respond to specific microbial threats.

**Figure 5:**
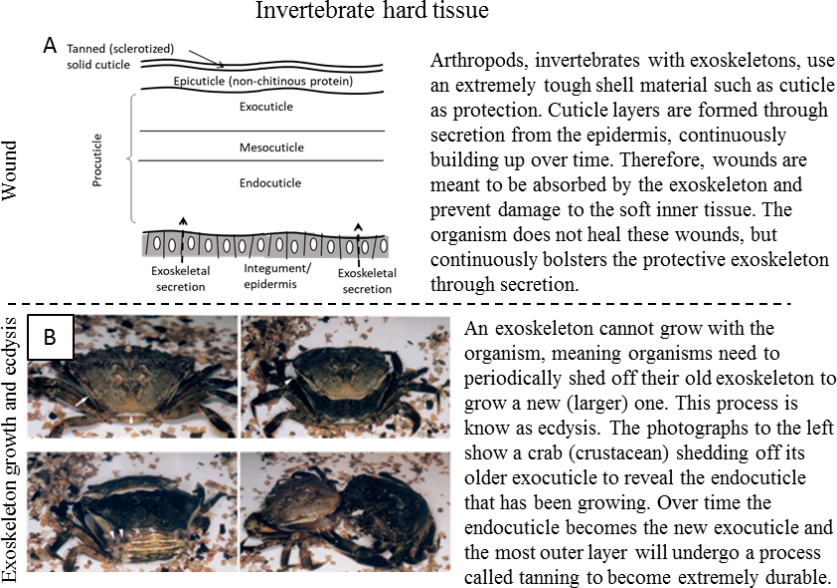
Healing response in the hard tissue (exoskeleton) of invertebrates to (A) wounds and (B) shedding. (A) The exoskeleton of invertebrates consists of several layers being secreted from the epidermis on the inner side. (B) The exoskeletons of arthropods cannot grow with an organism and are periodically cast off in a process known as ecdysis to reveal new exoskeleton growth. Reproduced with permission from [69], copyright 2000 The Company of Biologists.

**Invertebrate hard tissue:** Whereas vertebrates use an endoskeleton for structural support and dermal layers for protection, invertebrates known as arthropods use an exoskeleton. Arthropods are the most diverse grouping of animals, making up approximately 85% of known animal species. Exoskeletons form over a single layer of epithelial cells called the integument [66]. This underlying epidermis secretes skeletal material from which the exoskeleton builds. Exoskeletons are formed from a variety of material, but most typically from chitin, cuticle (a chitin–protein composite material), or calcium carbonate [67]. Figure 5A shows the various layers that compose the epidermis and exoskeleton [68]. Secretion of exoskeletal material adds to the exoskeleton’s thickness, growing the exoskeleton from within [26]. This very strong, protective layer does not detract from movement or flexibility, one of the reasons that arthropods are so successful in biodiversity [25]. Because the exoskeleton builds from within, damage to outer layers does not affect the growth process. In effect, a wound (scratch) that does not penetrate into the soft tissue is ignored by the organism as it does not affect their survival or behavior. The natural growth of the exoskeleton and periodic shedding renew its protective characteristics.

To sum up, invertebrates with an exoskeleton (arthropods) use a very hard protective layer to prevent damage to the softer inside tissue. This hard shell is continuously secreted and built up from within.

*Exoskeleton growth and ecdysis* – The robust exoskeletons of arthropods, while protecting the organism, also limit their potential for growth. Therefore, periodic growth cycles require an organism to shed off this hardened outer layer to allow for growth. The shedding process in a crustacean can be seen in Figure 5B, where the crab separates from its former shell, revealing new growth underneath [69]. This shedding process, similar to molting, is known as ecdysis in arthropods. As mentioned in the previous section, exoskeleton material is secreted onto the inner surface of the exoskeleton from the cellular epidermis. Using cuticle as an example of exoskeletal material, the mechanism for growth is for these cuticle secretions to form layers differing in rigidity and maturity. Once the inner layers are strong enough to support the organism, the old exoskeleton, formed of epicuticle and exocuticle, is shed away. The separation of the old exoskeleton is created through movement or by swallowing air to increase pressure on the rigid outer cuticle layers. After separation of the old exoskeleton, the (new) epicuticle then hardens through a process known as tanning [17].

To sum up, the exoskeleton, while protective, also limits growth in invertebrates. Therefore, organisms limited in their growth need to periodically shed off their exoskeleton to allow for growth. They accomplish this by growing a new exoskeleton from within and eventually forcing the old exoskeleton to separate and shed away.

**Figure 6:**
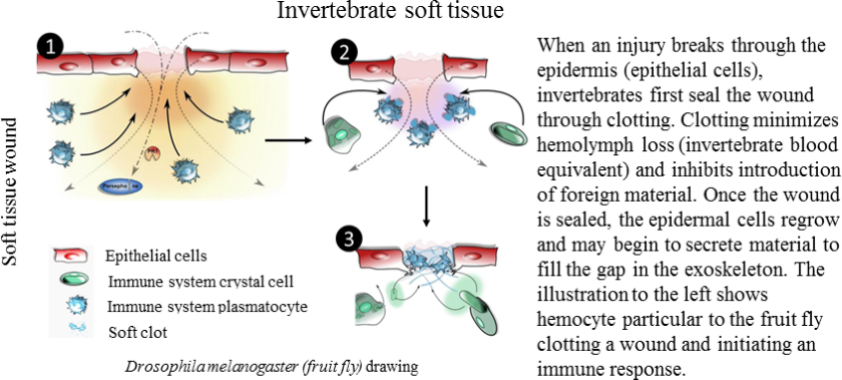
Healing response to invertebrate soft tissue wounds. Invertebrates rely on quickly clotting wounds to stem loss of hemolymph, which can quickly become fatal in their open circulatory systems. Adapted from [47].

**Invertebrate soft tissue:** Invertebrates heal soft tissue in much the same way as their vertebrate counterparts. This soft tissue healing response occurs in organisms without a skeletal system or in those with an exoskeleton when the wound is deep enough to pass through the exoskeleton and integument. Figure 6 shows the short-term and long-term healing stages in invertebrates [47]. The immediate need following an injury is to stem the flow of hemolymph, the equivalent of blood in invertebrates. Due to the open circulatory nature of most invertebrates, any hemolymph loss can quickly become fatal [16,27]. The stoppage occurs through clotting pathways including protein aggregation, cellular adherence, or cellular secretions at the wound site [70]. Once the hemolymph loss has subsided, epithelial cells repair the integument of the organism. Similarly, if an exoskeleton is present, the newly repaired epidermis will begin secreting exoskeletal material and patch holes in the exoskeleton.

To sum up, in invertebrate soft tissue wounds, the immediate need is to stop the loss of hemolymph through a number of clotting mechanisms. Once clotting has stopped the outflow of hemolymph, epithelial cells reform the integument, which can then return to normal function and secrete exoskeleton material if needed.

#### Flora

Like fauna, flora encompasses a great deal of variation. Therefore, choosing a way to group and describe healing mechanisms in an organized manner is important. The largest differences in healing occur between herbaceous and woody plants. Herbaceous plants are those that die down to ground level each year and then grow again. This includes perennials, biennials, and annuals. Annuals are plants that complete their entire lifecycle in a year, and perennials/biennials regrow from a part of the plant that stays alive but submerged during part of the year. Woody plants refer to those plants that stay alive above ground each year and are characterized by the wood tissue that continually grows on them. These plants include trees, shrubs, and lianas (woody vines). Woody plants tend to be more complex, with their yearly growth giving them a layered (e.g., tree rings) design. So, in addition to the healing and defense mechanisms common to all plant species, woody plants have more healing and defense options. Referring to Table 1, we first cover the healing and defense mechanisms that are common to all plants, both herbaceous and woody. All plants have cell walls, heal wounds, can have secretion cells, can use abscission, can have functional surfaces, and have immunity. These topics will be covered in a combined herbaceous and woody plants section. Next we look at those mechanisms that are particular to woody plants due to their added complexity. To review, the second column in Table 1 (Physical change) describes the physical changes that plants may go through after being wounded, with the third column describing the healing response and the fourth column describing the mechanism present in that response.

**Figure 7:**
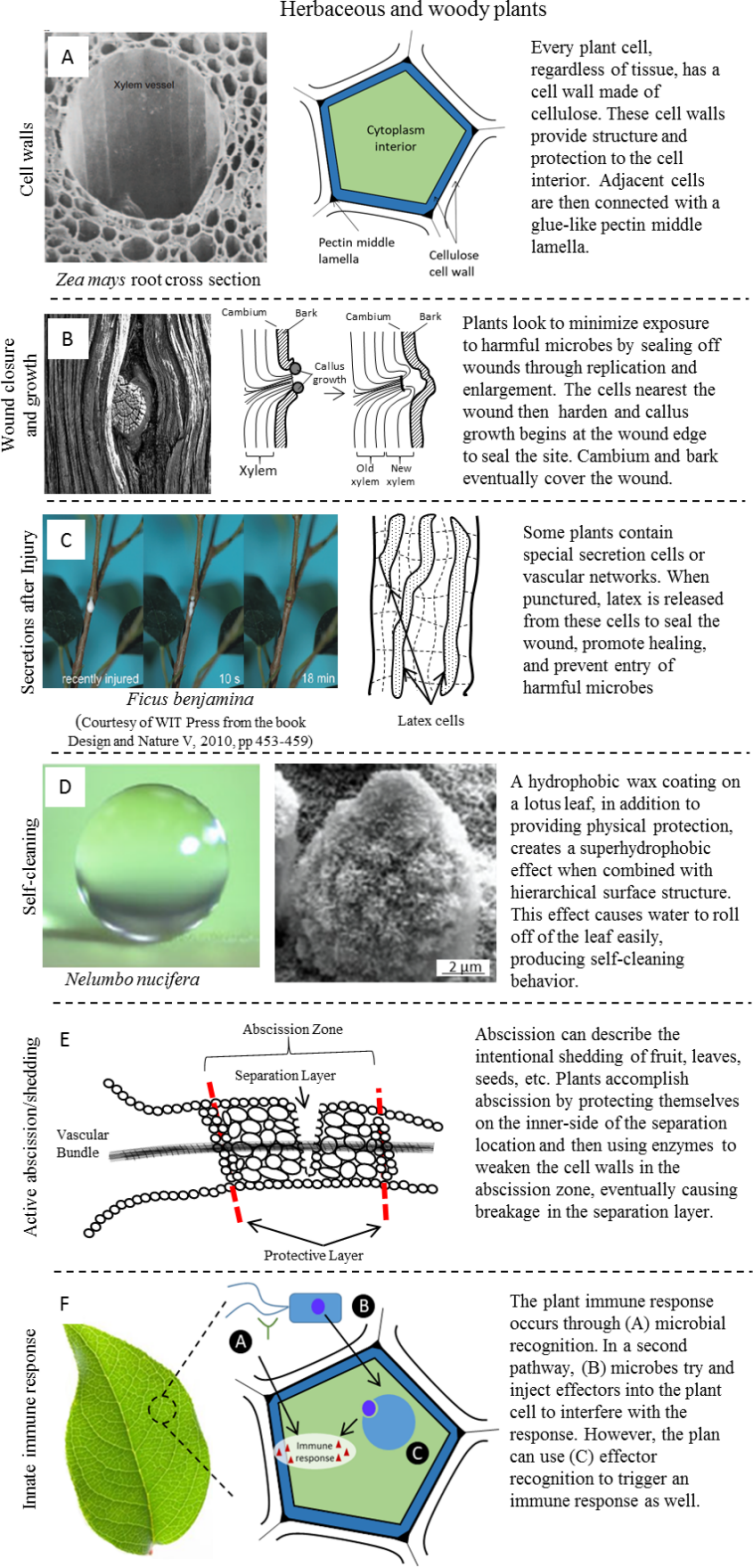
Healing and defense mechanisms in all plants (herbaceous and woody), including (A) protective cell walls, (B) wound closure and growth, (C) secretion cells, (D) functional layers, (E) abscission, and (F) immune response. (A) All plant cells are surrounded by a cell wall that (among other functions) protects the cell and provides structure. Left image reproduced with permission from [71], copyright 2005 Nature Publishing Group. (B) Cells at the surface of a wound in plants replicate and/or grow (hyperplasia and hypertrophy) to seal off the open wound and prevent disease. Left image reproduced from [72]. (C) Some plants have special secretion cells that release their contents to aid in healing and/or defense once the cell wall is damaged. Left image reproduced with permission from [73], copyright 2010 WIT Press. (D) Using a combination of hierarchical roughness and surface chemistry, plants can achieve complex self-cleaning behavior in surface (functional) layers. Images reproduced with permission from [32], copyright 2009 The Royal Society. (E) In order to purposefully release tissue such as damage leaves or seeds pods, plants use enzymes to break down tissue causing it to break more easily in a process called abscission. (F) Plant immunity relies on physical barriers in the cell wall and epidermis, but also uses an intracellular response to stave off harmful microbe attacks.

**Herbaceous and woody plants:** On the most basic levels, herbaceous and woody plants are comprised of the same basic materials and behave in a similar fashion to heal and/or defend themselves. This section looks at those healing and defense mechanisms common to all plant life, both herbaceous and woody. Figure 7 displays these common mechanisms in plant systems including cell walls, wound response, secretion cells, functional layers, abscission, and immune response.

*Cell walls* – The cellular building blocks of plants differ from their animal counterparts in that every plant cell is encased in a cell wall. The cell wall serves a variety of purposes in a cell including protection, structure, filtering, differentiation, water movement, and intercellular responses [71]. The structure of the cell wall can be seen in Figure 7A(a). Cell walls are formed from complex polysaccharides, cellulose and pectin in the case of land-based plants. Adjacent cellulose walls of different plant cells are “glued” together by a pectin middle lamella. Plants grow based on a system of selective division and growth as cells are stationary once formed. For example, Figure 7A shows the xylem and surrounding tissue of *Xea mays* [28]. In this picture, each of the compartments (xylem included) has formed from a single cell to fulfil a need within the plant. The meristematic plant cell is the equivalent of an undifferentiated stem cell in animals. The central xylem in *Xea mays* in Figure 7A(a) has grown to 30,000× its meristem size [71]. These cells grow to become whatever tissue the plant requires, including cells in the stems, roots, flowers, and leaves.

To sum up, the most basic unit of a plant, the plant cell, matures from a meristematic cell to become any tissue needed by the plant. However, all plant cells share the common characteristic of having a cell wall (joined by pectin interlayers) which provides structure and protection to the inner parts of the cell.

*Wound closure and growth* – As with animals, the immediate goal of plant healing is to minimize damage to give the plant the best chance at survival. As a plant cannot “bleed out” similar to an animal, the biggest threat to plants from an open wound is the introduction (influx) of harmful microbes or organisms. Reestablishing the integrity of the exterior of a plant is the top priority, so the cells at the wound site seek to close off access to the plant interior. The lack of blood or hemolymph removes the need for clotting to prevent any loss of liquids, so the cells at the wound use hypertrophy (increase in cell size) and/or hyperplasia (an increase in cell number) to close off the wound. After the wound gap has been filled, the cells at the wound surface harden to form a protective layer, reestablishing the protective integrity of the plant as a whole. Later on, cells at the edge of the wound overtake those which have hardened and died to desiccation at the surface [36]. Figure 7B shows an example of wound healing where a tree limb has been removed [72]. The cells at the wound (cut) harden to seal off the tree. After a period of time, the callus growth tissue overtakes the wound as it grows from the wound’s edge, creating the recognizable look in a tree knot [29].

To sum up, the biggest threat to a plant after a wound is the potential introduction of harmful organisms and microbes. In order to protect itself, the cells at the surface of the wound replicate or grow and then harden to block off access to the plant’s interior. The living tissue at the edge of the wound then slowly overtakes the wounded tissue.

*Secretions after injury* – Some plants have special secretion cells or vascularized networks running throughout their tissue, releasing their contents when punctured as a defense mechanism, to repel predators, and/or to quickly heal injuries [30–31]. Figure 7C shows an example of a weeping fig tree (*Ficus benjamina*), a latex producing tree known as a lactifer. Other plants may use volatile oils or gums for defense as well. These vascular cells or networks grow as the plant grows. Should an injury pierce through the cell wall of the secretion cell, the fluid encased within is released. Looking back to Figure 7C, one can see the drop of latex being released at the site of the cut to the stem of the tree [72–73]. This outflow of material both prevents harmful organisms from entering the plant and also dries quickly to seal off the wound, giving the plant an excellent chance to minimize damage and survive [74].

To sum up, some plants have developed special secretion cells or vascular networks to release fluid if an injury breaks its cell wall. The release of these fluids can provide a variety of desirable responses including quick healing upon drying or to ward off predators. The efflux, outward flow, of material from the wound also prevents microbes from being able to enter the main body of the plant’s interior.

*Self-cleaning* – As plants lack the ability to move, some abilities need to be built in. These types of surfaces, dependent on morphology and chemistry, are known as functional layers [28]. One prominent example of such behavior is the ability to self-clean, as can be seen in the lotus leaf in Figure 7D. The surface of the leaf causes water to roll off rather than slide due to the superhydrophobic functionality of the surface. In doing so, the rolling drops pick up or absorb particulates and dirt, cleaning the leaf. The superhydrophobic behavior is caused by a combination of hierarchical surface roughness at the nanometer and microscale combined with an epicuticular wax coating [32]. The replenishable wax coating, secreted by the epidermis at the surface of the leaf, offers another layer of protection. Maintaining a clean surface makes the plant operate most efficiently and can also remove harmful microbes, offering the plant the best chance for survival.

To sum up, some plants have built in functionality to their surfaces to accomplish complex tasks without the need for movement. These replenishable surface layers consist of combinations of hierarchical morphology and/or chemistry to create different wetting behaviors and are referred to as functional coatings. In the well-known case of the lotus leaf, the combination of hierarchical morphology and a wax coating makes the surface superhydrophobic and self-cleaning by causing water drops to roll off, rather than slide.

*Active abscission or shedding dead tissue* – A key part of a plant’s ability to survive and replicate is through its ability to remove damaged tissue and to drop seeds and replicate [33]. Because plants cannot move, another mechanism has evolved to remove material. Plants use abscission, a process of weakening part of a stem to cause it to break. After deciding the location of the break, known as the abscission zone, the plant prepares the inner side of the branch or stem as it would for a wound by sealing it off in a protective layer. Figure 7E shows the relationship in location between the abscission zone and protective layer that will be exposed once the stem or branch detaches from the plant [72]. While the protective layer forms, enzymes are released into the abscission zone to biochemically break down the pectin lamella that binds adjoining cell walls together. This degradation weakens the branch or stem and causes it to break, detaching whatever material lies on the far side of the abscission zone. Similar to a functional coating, this process lets a plant simulate complex behavior without the need to move.

To sum up, in order to remove material from itself or to drop seeds, plants have developed the ability to weaken stem and branches allowing them to break more easily. They accomplish this by protecting the part of plant that will be exposed (protective layer) while also releasing the enzymes to weaken tissue in the abscission zone through enzymatic degradation.

*Innate immune response* – Plants have an immune response similar to their animal counterparts. The first layer of protection is the innate protective layers that exist in the epidermis, functional coating, and secretions that keep harmful microbes away. Should a microbe infect a plant, the plant immune response is intracellular (within each cell) and is comprised of a two-part pathway, as shown in Figure 7F. In the first pathway, the plant uses microbe receptor recognition to initiate an appropriate cellular response to resist, kill, and/or remove harmful microbes. In response to the first pathway, microbes often release effectors (proteins) to try and trick the cellular response and ensure their own survival or to enhance their chance of success of infecting a plant cell. In this complex interaction, however, plants have also evolved an effector recognition system to counteract the effectors as well [34].

To sum up, plant immunity utilizes innate protective layers and fluids to prevent entry to harmful microbes. Should they be infected, plants also possess multistage cellular responses to recognize and react to these microbes.

**Woody plants:** The following two entries apply only to woody plants, whose seasonal growth, size, and complexity has allowed them to develop additional defense and healing mechanisms. To review, woody plants entail those plants that survive above ground all year, encompassing trees, shrubs, and lianas (woody vines). Wood, the characteristic tissue in these plants, is fibrous cellulose composite. Primary growth refers the elongation of plant tissue that is seen across most plant species. While some herbaceous plants undergo secondary (widening) growth, the process is mainly associated with woody plants. Secondary growth refers to cell division to gain girth across a plant, for example, a tree getting larger each year. In trees, this growth mainly refers to the cork cambium tissue in the epidermis and the vascular cambium (cylindrical source of undifferentiated meristem cells). These two tissues are known as the lateral meristems, and this yearly growth is the cause of tree rings. These growths also result in the growth of bark and the ability to form internal divides within the tree to seal away damage.

**Figure 8:**
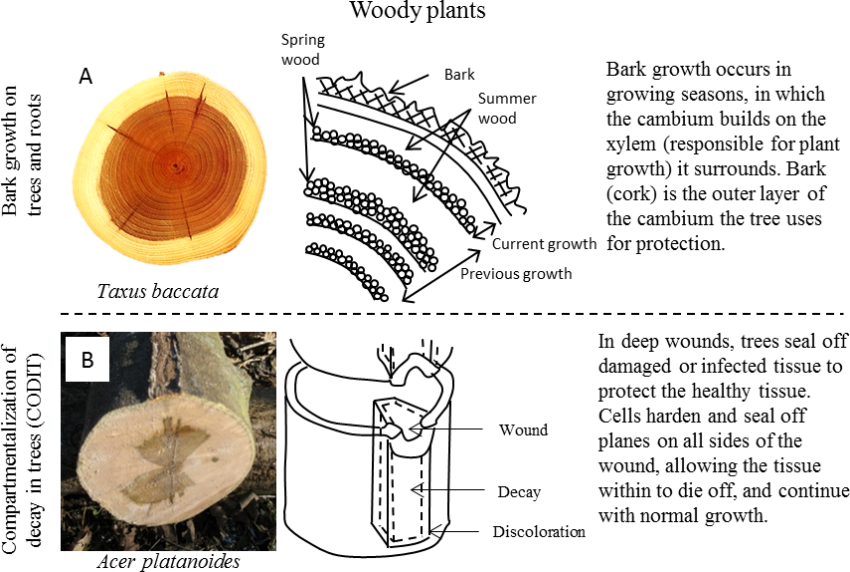
Healing and defense in woody plants, including (A) protective bark and (B) compartmentalization of decay in trees (CODIT). (A) Trees’ first line of defense is the hard outer bark that prevents physical damage from reaching the softer inner tissue. Left image reproduced from [75], copyright 2004 under CC-BY-SA 3.0 license (https://creativecommons.org/licenses/by-sa/3.0/). (B) Trees may aim to contain damage from infection and disease rather than heal it by sealing it away within natural barriers formed in the tree during growth. Left image reproduced from [76], copyright 2012 under CC-BY-SA 3.0 license (https://creativecommons.org/licenses/by-sa/3.0/).

*Growth of bark on the tree (epidermis) and roots (rhizodermis)* – The outermost portion of epidermis in trees, bark, is a hard, protective layer specific to woody plants. Bark, a general term, encompasses all of the tissue outside of the vascular cambium (cork, cork cambium, phelloderm, and secondary phloem), as seen in Figure 8A [75]. However, several other layers exist with a specific purpose, such as water retention or providing meristem cells [28,35–36]. The source of protective bark is similar to an exoskeleton because bark originates from inner growth. This outermost layer typically thought of as bark consists of dead tissue that has lost access to the nutrients and water from vascular system of the tree. This tissue dies and hardens, creating the protective outermost layer. The living inner layer continues to grow, providing a source for continuous bark production. As the tree grows, the bark cannot grow with it, causing the traditional thick, split look of bark.

To sum up, trees protect themselves using bark, a durable, hard material. Bark formation is a continuous process of growing from within, providing a barrier to the softer inner tissue of the tree.

*Compartmentalization of decay in trees* – Trees have a unique ability to seal off damage in infected areas, sacrificing part of themselves to resist the spread of decay for survival. The process is known as compartmentalization of decay in trees (CODIT). As previously described, the seasonal secondary growth of trees provides for a series of tissue growing on tissue. Tree growth is a largely compartmentalized and segmented process as each new season of growth envelops the last season’s, resulting in tree rings. Trees make use of these natural boundaries to form microbial barriers when necessary. Cells at the boundary change to form chemical and physical “walls” to prevent the spread of disease and/or decay. In a healthy tree, the parenchyma creates antimicrobial phytoalexins. When cellular signals call for CODIT, the tree uses these chemicals to form thin, chemical boundary layers to control the spread of disease and/or decay in a cylindrical shell segment of the tree trunk [37–38]. Figure 8B shows CODIT, where a segment of the tree has sealed off disease (darkened portion of the trunk), thereby protecting the rest of the tree’s tissue [76].

As with abscission, this ability highlights a major departure in the healing approach of animals. A lost limb in an animal may severely limit their ability to survive whereas a lost branch (or sealed away part of the tree trunk) does not have a huge effect on the chances of survival in a tree.

To sum up, one response to disease in trees is for the tree to limit and contain the damage rather than to try and heal it. Using natural boundaries formed during tree growth, the tree forms chemical and physical barriers to decay, protecting the remainder of the tree.

**Section review:** In this section we have looked at the methods used for defense and to prevent or heal damage in plants and animals. We have learned that types of defense or healing mechanisms are common to different types of animals, common to different types of plants, or common to plant and animal organisms in general. The need to provide homeostasis has been a common theme in animals, showing the need for an immediate (short-term) clotting response in stemming blood/hemolymph loss. Only after this process ensures survival will the body focus on repair and restoration of form and function to the body. A parallel ability seen in some plants, not to keep fluid in but to keep microbes out, is the vascular response to release fluid at the wound site in order to prevent infection, stave off predators, or speed up healing.

Throughout all organisms, healing has been shown to be a combination of the ability to prevent damage (innate protection) and to deal with harmful microbes when necessary through a cellular or humoral response. Furthermore, natural replacement of barriers, such as skin, hair, bark, and exoskeleton maintains a healthy exterior in addition to a strong defense. Within the plant kingdom, advanced actions such as cleaning and shedding have been replicated through alternative methods such as the use of functional surfaces and enzymatic degradation.

In the next section, we select prevalent examples of each of these healing and defense types and take a more mechanistic view of their processes.

### Prevalent self-healing mechanisms

**Figure 9:**
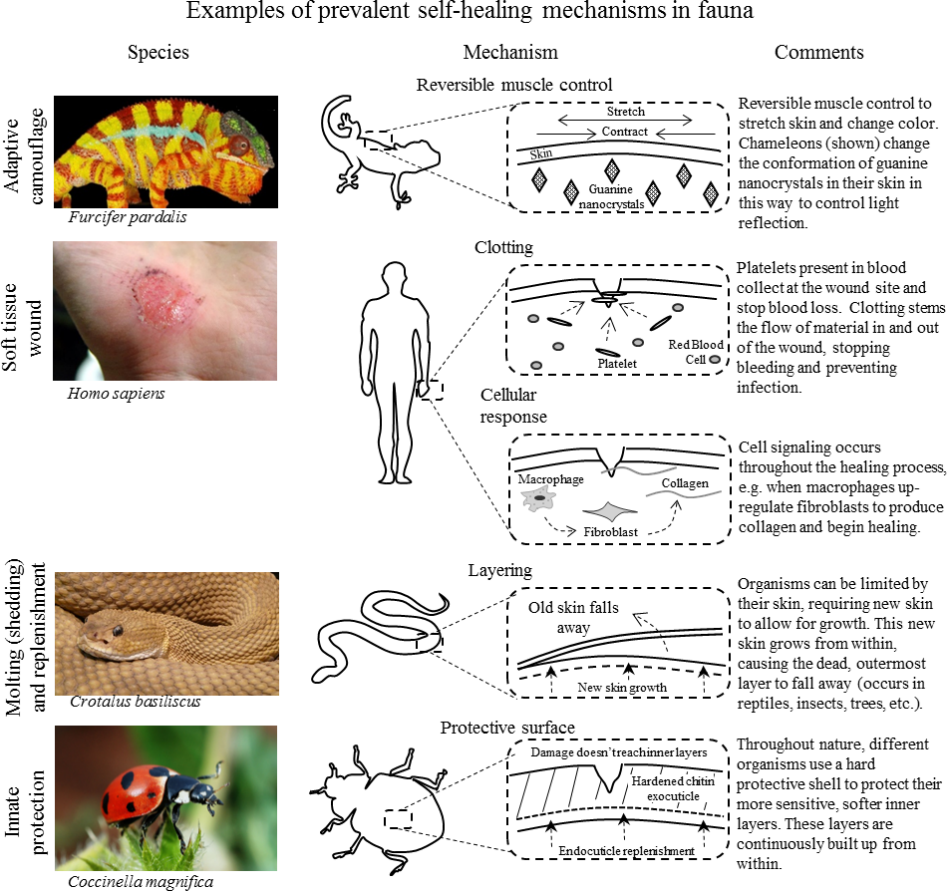
Examples of prevalent self-healing mechanisms found in fauna showing reversible muscle control in chameleons, clotting and cellular responses in humans, shedding in snakes, and innate protection in ladybugs. Each example is given with an illustration and description of the overall mechanisms. The top left image was reproduced from [15]. The second image on the left was reproduced from [7]. The bottom left image was reproduced from [77], copyright 2009 under CC-BY-SA 2.0 license (https://creativecommons.org/licenses/by-sa/2.0/).

**Figure 10:**
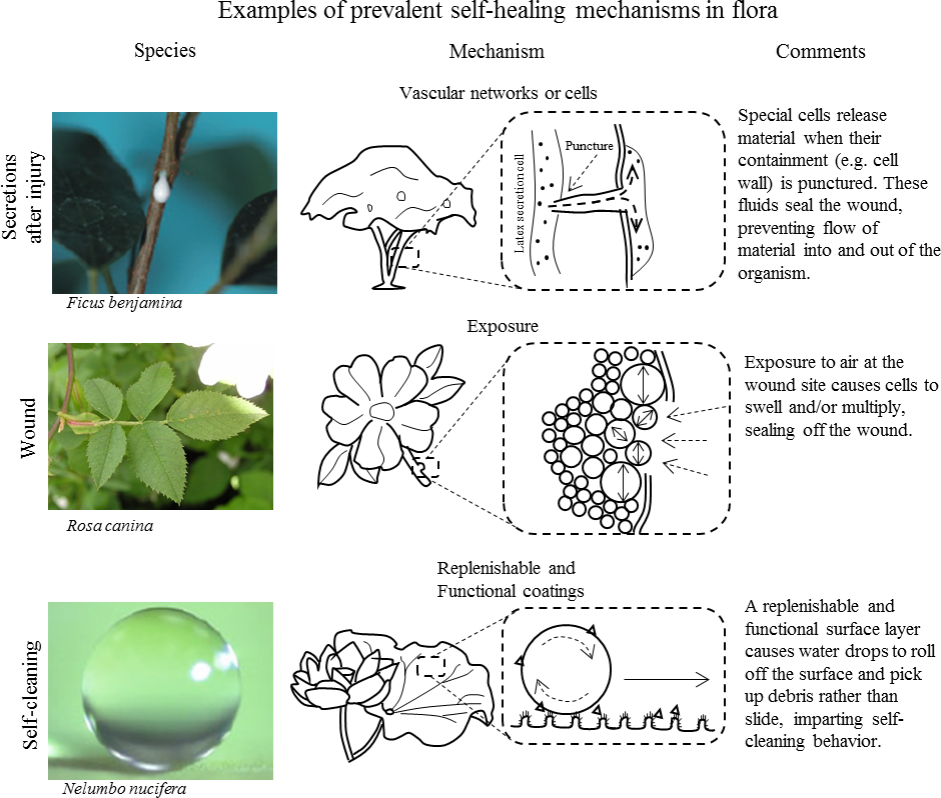
Examples of prevalent self-healing mechanisms found in plants showing vascular networks and cells of latex trees, wound healing through exposure in roses, and replenishable and functional coatings of lotus leaves. Each example is given with an illustration and description of the overall mechanisms. The top left image was reproduced with permission from [73], copyright 2010 WIT Press. The middle left image was reproduced from [78], copyright 2005 Michael Becker under CC-BY-SA 3.0 license (https://creativecommons.org/licenses/by-sa/3.0/). The bottom left image was reproduced with permission from [32], copyright 2009 The Royal Society*.*

After analyzing the healing and defense examples found in living nature, prevalent mechanisms are identified by looking at each example at its most basic level. Simplifying each type of healing and defense allows the basic mechanistic themes to be identified. For example, many types of healing and defense count on barriers to prevent harm such as exoskeletons, innate immunity, and tree bark. Others count on stopping of loss of fluids (blood or hemolymph) in the near term. These prevalent mechanisms are protective barriers and clotting, respectively. In this section, we identify eight such common mechanisms. We then look at them with a more mechanistic view of their overall function. While divided into prevalent examples from fauna and flora, summarized in Figure 9 and Figure 10 respectively, some of these mechanisms transcend across the plant/animal divide. The first column in each row shows a representative organism that displays the ability being described. After each picture of a representative organism, we present an illustration of the prevalent mechanism. Last, a description of the mechanisms in the expanded portion of the illustration is given.

#### Fauna

**Reversible muscle control:** Returning to the definition of healing as “returning to an original state,” the concept of reversible/replenishable systems come to the forefront. Muscle control offers a system that accomplishes the task of changing states reversibly and repeatedly over a lifetime. The most basic abilities of animals rely on this principle, including movement, breathing, and digestion. Muscle control can be either voluntary or involuntary, but both use the same mechanism of turning chemical energy into mechanical movement. Adaptive camouflage of chameleons is used as an example (Figure 9) due to its ability to control a physical characteristic of the animal. Chameleons use muscles to move the guanine nanocrystals in their skin and produce different colors. This reversible ability represents an advanced form of voluntary muscle control. To sum up, muscles represent a way to reversibly deform tissue using chemical energy.

**Clotting:** In looking at examples of vertebrates and invertebrates, the ability to quickly seal off wounds shows up repeatedly. The mechanism used to stop material flowing out of animals is clotting. In the blood containing closed circulatory systems of vertebrates or in the hemolymph containing open circulatory systems found in invertebrates, the ability to stop fluids from exiting their system is crucial for short-term survival following an injury. Although the actual material used to form clots may include cells, proteins, or other materials, the mechanisms of clotting relies on material aggregation at the wound site to plug the wound and stop material from flowing out of the body. A soft tissue wound to the hand is shown in Figure 9, with the top illustration showing the short-term clotting response. Platelets aggregate at the source of the wound, eventually stopping blood flow and maintaining homeostasis. A secondary effect of clotting is prevention of any harmful microbes from entering the body. To sum up, clotting is a mechanism seen throughout the animal kingdoms that relies on material aggregation at the source of the wound to provide an immediate healing response and stem the loss of fluids from a body.

**Cellular response:** In the broadest of the mechanisms, a cellular response refers to a body’s use of biochemical signaling pathways to initiate an appropriate response. This mechanism encompasses signaling from an immune response in vertebrates (shown) to enzymatic weakening of cells in the active abscission zone in plants. This universal ability across all organisms could be argued to be so overriding that it applies to every category. However, in this list of mechanisms, we aim to single out the ability of cells to initiate action in other cells such as secrete materials, attack the correct microbes, or bring another cell type to the area through biochemical signaling. The immune response to a hand injury is used in Figure 9 as a complement to the more short-term clotting response. In this example, macrophages up-regulate fibroblasts to produce collagen and begin healing. However, this example does not show the many parallel, simultaneous cellular pathways that are present such as immune response or pain suppression. To sum up, the ability of cells to specialize and communicate with each other gives all multicellular organisms the ability to form complex systems and provide complex healing responses.

**Layering:** The mechanism of layering is common throughout the animal kingdom. The relationship between layering and protective surfaces will be discussed in the following section. Layering has been singled out due to the mechanism of continual buildup from within and periodic shedding. In this light, layering allows organisms to replenish a damaged surface by casting it away periodically over a lifetime. In Figure 9, a molting snake is given as an example. The inner growth of the snake’s dermal layers forms beneath the older outermost skin. Once the inner skin is ready, the snake casts off the older skin to reveal a new, healthy body. To sum up, every animal has some outer tissue layer (epidermis), but some organisms periodically shed it away to allow for growth and maintain their health.

**Protective surface:** All organisms have some outer tissue layer, but some have evolved to have a very tough protective layer. This protective surface is meant to absorb damage that may otherwise prove fatal to softer tissue. Similar to layering, but even more widespread, this mechanism is seen in plants (trees), vertebrates (fish), and invertebrates (arthropods). This incredibly durable method of protection has been one of the sources of arthropods’ biodiversity success [25]. Because arthropods make up some 80% of the animal kingdom, it may seem as though layering and protection are one in the same. However, organisms such as trees and turtles do not purposefully shed their protective surfaces. Figure 9 uses an arthropod example, showing a ladybug with its colorful exoskeleton [77]. This exoskeleton protects the soft body and open circulatory system of the ladybug. This protective shell grows continuously from secretions from the epidermis. To sum up, the use of protective layers built up from the inner surface is found throughout all types of organisms and has proved to be a very effective means to prevent damage to more vulnerable tissue.

#### Flora

**Vascular networks or capsules:** While animals fight to keep fluids in after an injury, some plants use an outward flow of material to prevent harmful microbes from getting in, to irritate predators, or to heal wounds more quickly. In this way, plants with these special secretion cells or vascular networks release their contents when pierced similar to blood flowing when one is cut. Figure 10 shows a weeping fig tree as an example. When wounded, latex flows from the wound to prevent microbial entry. This fluid also dries and hardens quickly to help seal the wound. To sum up, some plants use special cells spread throughout their tissue or networks running throughout their tissue to release their contents once their cell wall has been pierced.

**Exposure:** A plant’s immediate need following a wound is to seal off the site of the wound and prevent microbes from introducing disease. To accomplish this task, plant cells multiply and/or grow (hypertrophy and hyperplasia) to fill the tissue gap that the damage has caused. Once the gap has filled, the cells at the surface harden to create a barrier and are eventually overtaken from healthy tissue growing from the wound edge. Figure 10 shows an example of hypertrophy and hyperplasia in a rose plant after its epidermis has been breached [78]. The cells at the surface divide, swell, and eventually harden to close the wound and protect the plant. The mechanism of interest is the physical change in cells based on their proximity to the wound. Cells at the wound site undergo hypertrophy, hyperplasia, and desiccation in a series of healing stages and based on environmental exposure. Similarly in CODIT, the cells in a tree harden and form barriers when exposed to particular microbes. One could argue that this simply represents another form of cell signaling. However, the focus of this mechanism is the physical change that is produced as a cell is exposed to a certain environment. To sum up, an effective method of healing shown in plants is through the use of hypertrophy, hyperplasia, and CODIT when exposure to a different environment causes physical change in the cells at the wound surface.

**Functional coatings:** Functional coatings are an essential part of many plants due to their static nature. These types of surfaces are found throughout nature, in plants and animals alike. In plants, a functional coating allows them to accomplish complex tasks without a need for movement. Figure 10 shows a prominent example, the self-cleaning behavior of the lotus leaf. The functional layer uses both morphology and chemistry of the surface to accomplish its given task. In the example shown of the self-cleaning behavior of the lotus leaf, the combination of hierarchical roughness and a hydrophobic wax coating causes drops to roll off the surface rather than slide. The rolling action causes each water drop to “grab” debris and remove it from the surface. These replenishable wax layers are yet another layer of physical protection to the leaf, and the self-cleaning behavior of the surface allows the plant to maintain a clean, healthy surface. To sum up, functional coatings created through a combination of surface morphology and chemistry can be used to create complex behavior in static systems.

#### Section review

This section has categorized the prevalent healing and defense mechanisms described in the previous section. Various examples have been given in Figure 9 and Figure 10 to show the common mechanisms of healing and defense. The diversity of organisms in which these common mechanisms are seen speaks to their effectiveness at providing protection and healing. When looking at innovative design in new materials, nature is often used as an inspiration. Looking at the most effective mechanisms provided by nature is sure to provide insight for developing mechanisms that will be effective in synthetic materials as well. In this section, a great deal of information has been categorized, and the eight mechanisms that have been discussed represent nature’s best (evolutionary) methods of mitigating and healing damage. In the following section, we look at ways in which these mechanisms have been used to inspire new self-healing material designs.

### Types of bioinspired surfaces

**Figure 11:**
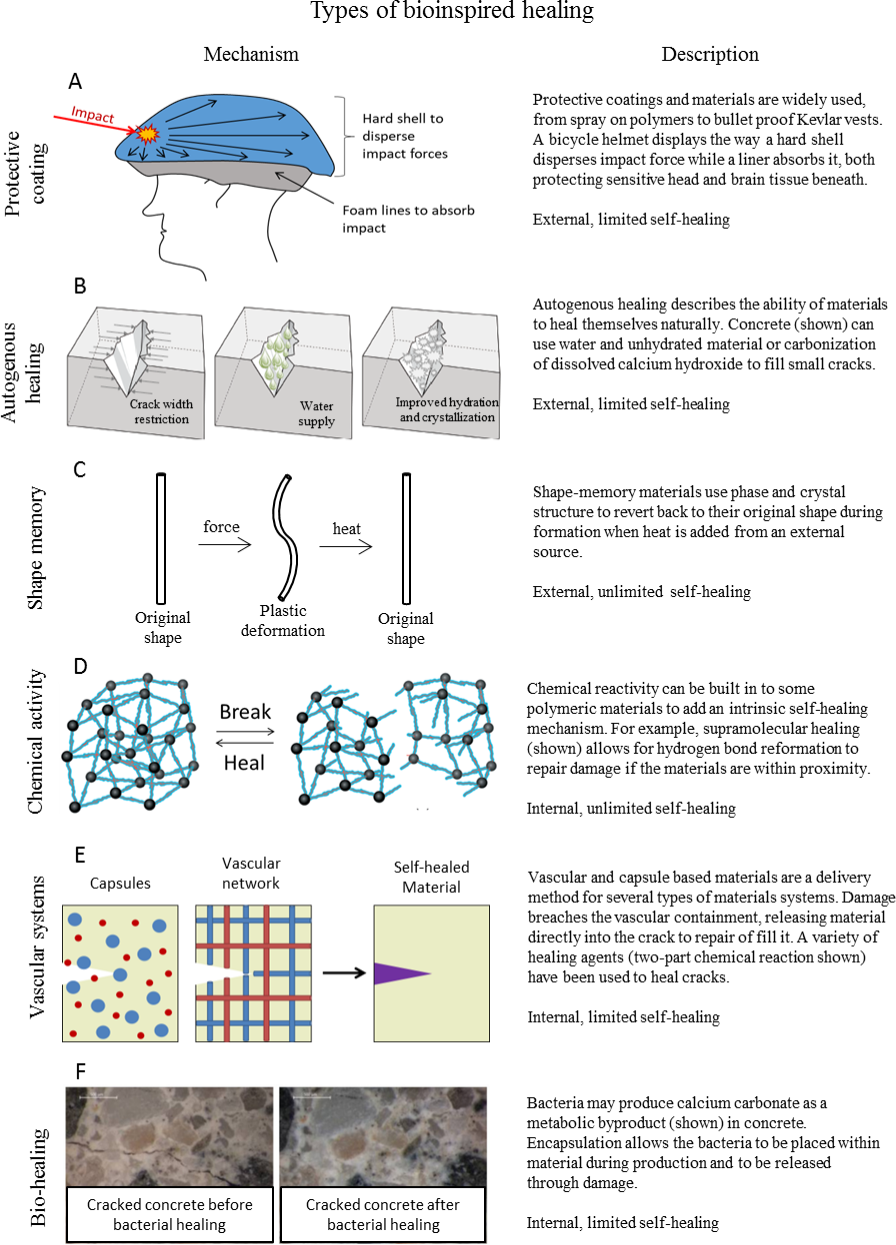
Types of bioinspired healing materials including (A) protective coatings, (B) autogenous healing, (C) shape memory, (D) chemical activity, (E) vascular systems, and (F) bio-healing. Each example is given with a description of the mechanism and comments labeling the (internal or external) source of healing materials/energy and the (limited or unlimited) nature of healing responses. The image in (B) was adapted from [79]. The image in (D) was adapted from [80], copyright 2013 RSC Publishing. The image in (F) was adapted from [81].

The mechanisms of the previous section have shown the prevalent healing and defense mechanisms found in nature. Figure 11 shows examples where these principles have been applied in bioinspired self-healing (synthetic) materials. The goal in creating self-healing materials is to remove the human element from the repair process. However, such a highly desirable outcome is limited by the ability to deliver the elements necessary to repair a material, mainly replacement material and energy, to the location where repair is necessary. The first column in Figure 11 provides an example of each self-healing material followed by a brief mechanism description in the second column. The “comments” column labels each self-healing material in terms of its ability to deliver those elements necessary to repair a damaged material.

As mentioned, the limiting factors in self-healing materials are the ability to deliver material and energy to the site of the damage. Therefore, the comments section labels each material at either having internal or external stimuli and as having either limited or unlimited healing abilities. Stimuli refer to the source of the materials necessary to heal. External examples include using a paint brush that requires material and energy external to the material or using a heat gun which uses only energy external to the system. Limited and unlimited refers to the number of times a material can heal itself. If a material can return to its original form and function an endless number of times, regardless of the stimuli source, then it is unlimited. Similarly, if a material heals itself with no external energy or materials (internal stimuli), but can only heal a finite number of times then it is limited. The ideal system would use internal stimuli and have unlimited healing abilities in order to remove any necessary outside intervention in returning it to full form and function.

#### Protective coatings

Protective coatings are based on the use of durable layers to protect more sensitive material beneath. Humans recognized this trick in nature quickly, as thick hides were used in ancient times as armor for weaker human bodies. Although the materials have advanced, the concept has remained. Figure 11A shows a bicycle helmet, which is used to absorb and disperse force generated in a crash in order to protect one’s head and brain, the most sensitive parts of the human body. Other examples include Kevlar bulletproof vests, spray on polymer coatings to protect furniture, and metal push bumpers on automobiles. With the growing field of carbon technology and composites, this area of research will continue to strive for stronger, thinner, and lighter methods of protection. A similar concept but different approach is the use of layering for protection, having a disposable panel that is more easily replaced then the whole, e.g., a car panel or the roof on a house. Materials such as these are not built to fail, but are much less costly to replace than the car or house they cover. When looking at protective coatings as a healing method, they are external and limited in nature.

To sum up, protective coatings are meant to take in damage rather than the sensitive or costly materials they protect, but they usually need to be reapplied or replaced afterwards.

#### Autogenous healing

Autogenous healing refers to a substances natural ability to heal itself and mainly refers to concrete. The autogenic healing of concrete was first noticed in Roman aqueduct design, where the inclusion of a lime component and volcanic ash would help cement the stones in place. In modern times, concrete is the most used construction material on Earth, making the addition of any self-healing characteristics very important both economically and in terms of infrastructure upkeep. De Rooj et al. [82] report two chemical self-healing behaviors in dealing with small cracks. Figure 11B shows the different ways in which concrete has autogenous behavior [79].

The first simply exists due to non-hydrated material in the concrete. When being applied, concrete is mixed with water and set to dry. Not all of the concrete comes into contact with the water, so some non-hydrated concrete dries into the concrete, which may then become exposed to water should a crack form nearby. The second method involves and aqueous formation of calcium carbonate crystals inside the crack [83]. Both of these healing pathways require water to be added to the system, making them require external stimuli. In outdoor uses, however, humidity or rain is readily available and can initiate the healing. The amount of healing material is limited, in that the reliance on non-hydrated materials or calcium ions going into solution (for calcium carbonate formation) cannot be endless in nature.

To sum up, autogenous behavior describes the ways in which concrete can heal itself after setting. While useful due to the scale of concrete usage, the behavior is limited and unreliable in the long term.

#### Shape memory

Shape memory materials are a type of material that can be reversibly and endlessly healed. This reversible behavior resembles muscle behavior in that both are reversible given an external energy source. Shape memory materials, typically polymers [84] or metal alloys [85], are cast into a permanent (stored) shape. This material can then be deformed, but made to return to the stored shape after some external stimulus is applied, typically in the form of heat. Figure 11C shows an illustration of this cycle of deformation and returning to a stored state with the application of external energy. In a more direct use of external stimuli to introduce energy in targeted areas, electromagnetic induction [86] and nanoparticles combined with oscillating magnetic fields [87] have been utilized as well. These materials can be recast to a new permanent stored shape, but typically at extreme conditions. For example, a nickel–titanium alloy, a common shape memory smart material, sets its structural memory shape when heated to 500 °C [88]. External stimuli in the form of temperature changes then use martensitic–austenite phase transitions to cause the material to return to reform to the structural memory shape after being plastically deformed. These types of materials do use external stimuli, but can return to their structural memory shape any amount of times, giving it unlimited healing ability.

To sum up, shape memory materials offer unique abilities to endlessly return to a stored state, but rely on the use of targeted external energy.

#### Chemical activity

Chemical activity is an umbrella term for materials that use equilibrium disruption, reactive species creation, or catalysis as ways to heal materials after damage takes place [89]. It is worth noting that chemical species are often paired with a delivery method such as vascular networks and capsules, which will be discussed. As standalone self-healing methods, these chemical mechanisms can be built into a material. Each of the three techniques has varying likenesses to nature, with equilibrium disruption most closely representing a mechanism that would be found in nature. On a more general level, they all rely on creating a chemical potential during the injury that will result in self-healing behavior as the system returns to equilibrium [90].

The limitation in building chemical activity into a material, typically polymers, is two-fold. First, within a polymer chain, cleaving the chain and hoping for repair requires the “correct locations” on either side of the damage to line up correctly to heal. This problem has been addressed by including functionality into side chains or by shortening the chain lengths in general in an attempt to have more “correct locations.” The second limitation is the reactivity and time component of the healing. The reactive species that are created need to stay stable until they come into contact with the other reactive species, which is not always a simple task. For example, radicals exposed to an open environment tend to react quickly. Equilibrium disruption is far less problematic in these areas, as reported by Diesendruck et al. [91]. Several examples of successful and repeated healing mechanisms are given. These examples include reverse Diels–Alder, Grubbs catalyst, and supramolecular forces [80]. Figure 11D shows an example of supramolecular forces. After being cleaved, the hydrogen bonds in the material move towards an equilibrium state by reforming the broken bonds, eventually causing the material to heal. In each of these cases, the mechanical division of the material tends towards a more stable “healed” product. Diesendruck et al. [91] point out the material property limitations of such materials, which are typically softer and low-modulus materials.

When analyzed for healing, introducing chemical activity to the site of a wound relies on localized response built into the material, making it an internal healing type. Similarly, the idea that the damage itself creates the reactive species means that these types of healing can theoretically be used any number of times. So, chemical activity seems like the ideal healing material, both coming from an internal source and being continuous in effect. However, one must note the limitations in the types of materials that these mechanisms are typically found in, softer polymeric materials. To sum up, chemical activity has the elements necessary for ideal self-healing materials (internal stimuli and unlimited healing cycles), but is limited to very particular types of materials that restrict possible usage.

#### Vascular networks or capsules

As mentioned in the previous section on chemical activity, delivery of reactive species creates issues in self-healing materials [91–92]. On one hand, one wants reactivity, but on the other hand one wants this reactivity to be localized (e.g., within the crack). One of the most promising methods to avoid these problems is through the use of capsules or vascular networks. This approach mimics the weeping fig tree releasing stored latex after its secretion cells are damaged during an injury (Figure 10). Figure 11E shows the two vascular systems, capsules and networks, which can be used to hold reactants separately until damage occurs. For example, reactants of a two-part epoxy can be encased individually in capsules throughout a material. When the material suffers damage, both types of capsules will be damaged and release their contents. These two parts will mix within the crack and harden to seal the damage. By using smaller capsules dispersed throughout the material in high concentrations, one can almost ensure that the site of the damage will breach the capsules of any number of reactants. Use of larger, continuous vascular cell systems increases the amount of material available at each site, extending the number of times a material can heal. By using a vascular approach one almost guarantees access to reactants or other materials needed at the wound site. Taking the vascular approach one step further, one may connect the vascular network to a continuous source, providing a limitless source of healing in theory as long as the network stays intact through the damage.

Healing using capsulized reactants or vascular networks answers many of the limitations found in other methods due to the separation of healing reactants from the material itself. This separation allows capsules to be included in any material in which it can withstand the production or mixing. Because the capsules and networks are located within the material, they are viewed as an internal healing source. However, the limitation lies in the number of times a material may heal itself. Capsules offer a one-time healing response. Vascular networks, by providing a larger supply that can flow to the site of damage, circumvents this limitation somewhat but still offers a limited number of healing responses. Last, connecting a vascular network to an external and unlimited source lets the material theoretically heal countless times, but changes the material from relying on only internal sources to relying on an external supply.

To sum up, vascular systems offer a great deal of promise by allowing use of chemically active species and ensuring their delivery at the site of the wound (that causes their release). The use of networks rather than cells promises to let materials have multiple healing cycles at a given location. However, in the long run these types of materials remain limited in healing cycles once their internal materials have been used.

#### Bio-healing

The final example of bioinspired materials is an extension of capsules, but is worth a separate discussion in that it utilizes the diverse ability of life to exist almost everywhere, use minerals as a food source, and create “useful bio-waste.” The use of bacteria has been successfully applied to concrete where capsulized bacteria are released at the site of damage as reported by Jonkers [93]. The bacteria, seen in Figure 11F, convert water and a mineral food source to create a desired waste product that fills in damage [81,94–95]. As mentioned previously, concrete is often exposed to the elements, providing the necessary water, and the food source may be capsulized or built into the concrete matrix. The bacteria multiply and feed, releasing calcium carbonate as a metabolic byproduct and sealing the crack. Similar to autogenous healing in concrete, this healing pathway requires water to be added to the system, making them require external stimuli. In outdoor uses, however, humidity and rain is very readily available and can initiate the bacterial reproduction. As a capsulized approach or vascular approach, healing can only take place a limited number of times.

To sum up, bio-healing is a unique approach to healing a widely used material in concrete, but retains the same issues as autogenous healing of limited and unreliable long-term usefulness.

#### Section review

In this section, we have outlined the most successful pathways that have been derived from biological sources. Looking at each type of self-healing, we analyzed the source of healing materials and energy as well as the ability to heal multiple times. Inspiration for these self-healing smart materials has been pulled from each of the prevalent mechanisms in nature seen in the previous section; using armor as protective sources and layering, looking to chemical activity and autogenous behavior similar to cellular responses, designing shape-memory materials that behave similar to muscles, and capitalizing on the design of vascular networks and capsules to deliver desired materials to a desired location similar to a secretion cell.

**Figure 12:**
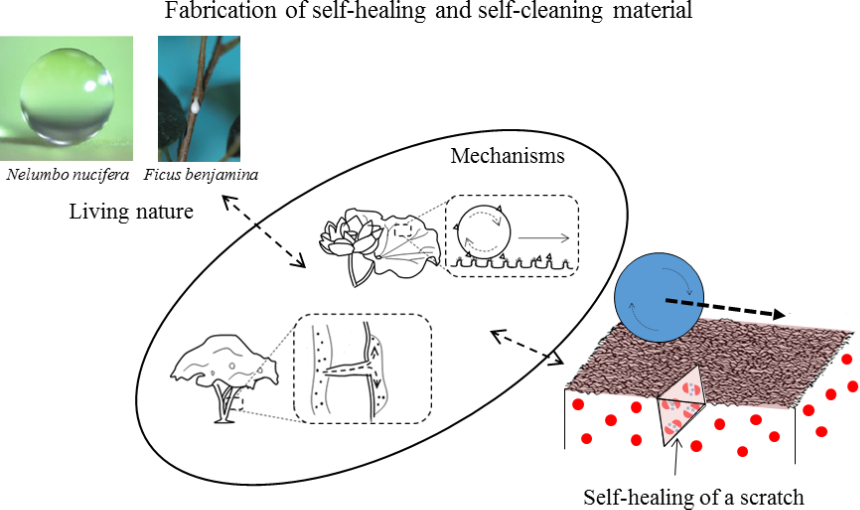
Translation of healing in nature into self-cleaning and self-healing materials. Mechanisms in nature, the self-cleaning lotus leaf and vascular cells of the weeping fig tree, can be translated into a new self-cleaning, self-healing material based on replenishable functional surfaces and targeted capsule delivery of self-healing materials that also preserve the functionality of the surface. Upper left images reproduced with permission from (left) [32], copyright 2009 The Royal Society, and (right) [73], copyright 2010 WIT Press.

In understanding and relating these biological mechanisms and self-healing materials, we can find gaps or new approaches to self-healing material design. Figure 12 illustrates an example of the type of opportunities that may arise with inspiration derived from the functionality of a lotus leaf and vascular cells of the weeping fig tree. In this case, replenishable functional surfaces combined with capsule delivery of healing materials creates self-cleaning, self-healing material behaviors. Should the self-cleaning functional surface be damaged, the targeted release of capsulized healing materials may be used to preserve functionality.

## Conclusion

In the current multidisciplinary scientific landscape, the borders between fields are being blurred constantly. This idea has been one of the overarching themes in biomimetic research and bioinspired design. Looking to nature, where evolution has engineered and optimized complex systems and structures over the course of millions of years, only makes sense. However, one of the limitations in this area has been the ability of a scientist in one field to fully categorize and digest the large and complex systems of information in another. When a scientist does achieve this level of understanding, the world often gains a novel material or interesting new approach to solving an old problem. In today’s world, self-healing materials are an interesting problem sitting at the border of botany, materials science, chemistry, biology, physics, mechanical engineering, and chemical engineering, just to name a few. Therefore, this review has aimed to create a map relating nature’s most successful healing and defense mechanisms to today’s most well-known approaches to self-healing materials.

**Figure 13:**
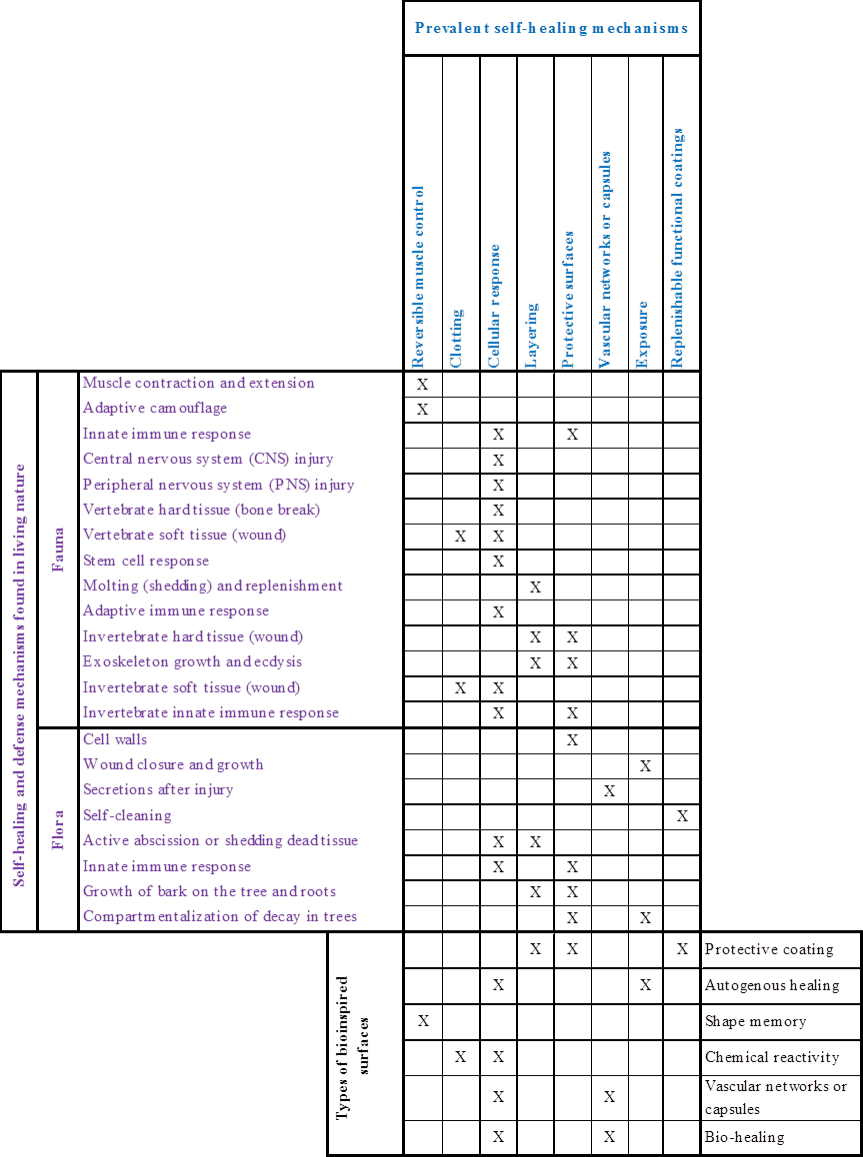
Chart relating self-healing and defense mechanisms found in living nature with prevalent self-healing mechanism categories with bioinspired types of synthetic materials.

We have learned some overarching themes of animal and plant behavior in the prevention (of) and response to injury. Analyzing these behaviors led to identification of major (mechanistic) themes in healing and defense found throughout living nature. Figure 13 displays the relationship between healing/defense mechanisms found in living nature and their larger categories. The prevalent mechanisms are reversible muscle control, clotting, cellular response, layering, protective surfaces, vascular networks and capsules, exposure, and functional coatings. These mechanisms have seen the most success through the lens of evolution and biodiversity, and their success speaks to their potential translation to synthetic materials, many of which have already been seen. Looking again to Figure 13, we relate the synthetic self-healing materials back to prevalent mechanisms seen in nature. For example, autogenous materials built into concrete rely on chemical reactions (cellular activity) that take place once the concrete is exposed to moisture in the environment (exposure). The question remains of how to use this new understanding of these complex relationships and mechanisms.

First, with healing in mind, we chose the evolutionary divisions that offered the best insight into healing and defense mechanisms in living nature, but other breakdowns do exist. For example, where we chose to look at vertebrates and invertebrates, another division exists in land-dwelling versus submerged species. The differences in their environments and behavior may offer additional insight. Regardless, the hope is that this overview provides a framework for relating behavior seen in prevalent mechanisms in living nature to bioinspired materials.

Second, we hope this review acts as a resource when looking into limitations and problems with existing materials. Over the past millions of years, nature has most likely run into similar problems and limitations, evolving to deal with them. For example, using chemically active species appears to be the best approach to providing intrinsic self-healing in materials. However, the problem with chemically active species is that one only wants them to be active with the correct materials or locations. Looking to nature, a parallel case can be found in trees where gums, latex, and volatile oils provide excellent healing and defensive capabilities to lactifer trees, but only if those materials are contained and released at the correct location. Nature solved this problem using special secretion cells to hold the material until needed. This approach has been used to design vascular networks and capsules to effectively deliver the chemically active species. Identifying parallel problems in nature may offer a unique approach to an existing problem.

Last, we hope that this information can open up new avenues of insight and research into self-healing materials. As discussed, in Figure 13 we relate existing materials back to the prevalent self-healing mechanisms in nature. However, one could argue that for every combination of prevalent mechanisms in nature, a possible approach to self-healing materials exists. For example, opportunities may lie in the combination of capsules with functional surfaces and emulsions that utilize micro- and nanospheres. Similarly, muscle control (shape memory) combined with layering could utilize UV-active smart surfaces that change shape periodically with the Sun to produce inflow and effusion of material necessary to produce layers. Only by understanding ideas and mechanisms across these borders of engineering and biological sciences can we start to take advantage of these types of opportunities.

Finally, this overview relating self-healing materials back to their biological roots will offer some insight into this crossover of interdisciplinary knowledge.
